# Cue-weighting in processing of prosodic boundaries in Dutch: An event-related potential (ERP) study

**DOI:** 10.3758/s13423-025-02843-x

**Published:** 2026-01-20

**Authors:** Jorik Geutjes, Rachida Ganga, Elanie van Niekerk, Victoria Reshetnikova, Aoju Chen

**Affiliations:** Institute for Language Sciences, Utrecht University, Trans 10, 3512 JK Utrecht, the Netherlands

**Keywords:** Prosodic phrasing, Processing, EEG, Event-related potentials, Closure positive shift, Dutch, Cue-weighting

## Abstract

Across languages, major prosodic boundaries, such as intonational phrase (henceforth, IP) boundaries, are typically signalled via three types of prosodic cues, namely pitch change, final lengthening, and pause. However, the relative weight of each cue in the perception of IP boundaries differs across languages. Little is known about which cues are important in Dutch. This study investigates cue-weighting during processing of IP boundaries in Dutch by examining the effects of varying combinations of cues on the neurophysiological correlate of boundary processing, i.e., the Closure Positive Shift (CPS). Twenty-five native speakers of Dutch listened to renditions of a name sequence, connected by the coordinating conjunction *en* (‘and’), i.e., *Moni en Lilli en Manu,* with or without an IP boundary after the second name. Event-related potential (ERP) results showed that the CPS was elicited by IP boundaries if a pause was present, regardless of whether pitch rise or final lengthening was omitted. In contrast, no CPS was observed when the pause cue was absent, even if both the other two cues were present. Together, these findings suggest that pause has a crucial role in the processing of IP boundaries in Dutch coordinated constructions, thereby differing from findings previously reported for the other West Germanic languages such as English and German.

**Supplementary Information:**

The online version contains supplementary material available at 10.3758/s13423-025-02843-x.

## Abstract (Dutch)

In verschillende talen worden prosodische grenzen, zoals grenzen van ‘intonational phrases’ (IPs), doorgaans aangegeven met drie specifieke prosodische aanwijzingen, namelijk toonhoogteverandering, finale verlenging en pauze. De relatieve bijdrage van elk van deze aanwijzingen aan de perceptie van IP-grenzen verschilt echter per taal. Over welke aanwijzingen belangrijk zijn in het Nederlands is nog weinig bekend. In deze studie wordt de weging van aanwijzingen tijdens de verwerking van IP-grenzen in het Nederlands onderzocht door de effecten te bestuderen van verschillende combinaties van aanwijzingen op de neurofysiologische reactie op grensverwerking, de Closure Positive Shift (CPS). Vijfentwintig moedertaalsprekers van het Nederlands luisterden naar verschillende versies van een gesproken naamreeks, verbonden door het nevenschikkende voegwoord *en*, bijvoorbeeld *Moni en Lilli en Manu*, met of zonder een IP-grens na de tweede naam. De ERP-resultaten lieten zien dat de CPS ontstond bij de verwerking van IP-grenzen wanneer er een pauze aanwezig was, ongeacht of een toonhoogtestijging of finale verlenging ontbrak. Daarentegen werd geen CPS waargenomen wanneer de pauze afwezig was, zelfs als beide andere aanwijzingen aanwezig waren. Samen wijzen deze bevindingen erop dat pauze een cruciale rol speelt in de verwerking van IP-grenzen in Nederlandse gecoördineerde constructies, wat afwijkt van eerdere bevindingen voor andere West-Germaanse talen zoals het Engels en het Duits.

## Introduction

Spoken language consists of utterances which can be segmented into smaller linguistic units, such as words and phrases. To convey this internal structure of utterances, speakers utilise the prosodic properties of the speech signal, including variations in pitch, duration, and intensity. Listeners use this information to group words together into prosodic units, known as *prosodic phrases*. Within utterances, intonational phrases (IPs) are generally considered to be the largest of these units (Gussenhoven, 2004; Ladd, 2008, cf. Grice 2006), largely corresponding to major syntactic constituents, such as clauses (Beckman & Pierrehumbert, 1986; Nespor & Vogel, 1986; Selkirk, 2005).

In addition to its utterance structuring function, prosodic information can help disambiguate utterances. For example, the sentence *John says Mary is smart* is structurally ambiguous, as the word *smart* could refer to either *John* or *Mary*. The sentence can be disambiguated by placing an IP boundary either directly after *says* or after both *John* and *Mary*, as illustrated in (1) and (2):

1. [John says]_IP_ [Mary is smart]_IP_
2. [John]_IP_ [says Mary]_IP_ [is smart]_IP_

The boundaries of IPs are marked by different segmental and prosodic cues, both phrase-initially and phrase-finally. For example, on the segmental level, IP boundaries can be indicated by glottalisation (e.g., Redi & Shattuck-Hufnagel, 2001) and phrase-initial strengthening (for a review, see Cho, 2016). On the prosodic level, the focus of the current study, there are three types of cues to IP-final boundaries: *pitch change, final lengthening*, and *pause* (e.g., Wagner & Watson, 2010). Pitch change involves a deviation from the default pattern of pitch declination over the course of an utterance. This change can be manifested either as a *pitch reset*, i.e., a suspended lowering of pitch preceding the boundary and an increase in pitch after the boundary, or as a so-called *continuation rise*, i.e., a rising preboundary pitch contour indicating utterance continuation (e.g., Chen, 2007). Final lengthening, also called prefinal lengthening, phrase-final lengthening, or preboundary lengthening, refers to the increase in syllable duration before a prosodic boundary (e.g., Cambier-Langeveld, 2000). Pause marks the IP boundary as a silent interval between IPs (e.g., Ferreira, 1993).

Although boundaries are typically marked by a combination of these three prosodic cues across languages, the relative importance of each cue differs between languages. For example, during offline perception in American English, listeners rely on the presence of pauses to detect boundaries in spontaneous speech (Mo, 2010) and in syntactically ambiguous noun phrases, such as *[turkey salad] [and coffee]* vs. *[turkey], [salad], [and coffee]* (Zhang, 2012). In contrast, Mandarin Chinese listeners rely primarily on pitch cues (Zhang, 2012; but see Yang et al., 2014), whereas German and French listeners depend on a combination of final lengthening and pitch change in offline boundary perception, albeit with considerable individual variability among speakers (van Ommen et al., 2020).

Little is known, however, about cue-weighting in perception of prosodic boundaries in Dutch, another West Germanic language widely studied for its prosodic features. Developmental research has, however, suggested that possible crosslinguistic differences in cue-weighting between Dutch and other West Germanic languages may underlie developmental differences in early word-segmentation ability. More specifically, Johnson and Seidl (2008) showed that Dutch-learning 6-month-olds could discriminate a word sequence with an intervening clause boundary from the same sequence without such a boundary only when it was cued by a pause, while English-learning 6-month-olds could do so in the absence of a pause (Seidl, 2007). They argued that Dutch-acquiring infants’ reliance on pauses may reflect Dutch-specific cue-weighting in adults’ perception of phrase boundaries. They further suggested that this reliance may lead to difficulties in detecting prosodic boundaries when pauses are absent, potentially explaining the observed delay in word segmentation in Dutch-acquiring 7.5-month-olds relative to English-learning peers (Houston et al., 2000; Kuijpers et al., 1998).

Despite these developmental findings, there is insufficient and unconvincing evidence to support the idea that the pause cue plays a crucial role in boundary processing for Dutch-speaking adults. Aside from the aforementioned study on early speech perception in Dutch-learning infants, there have been only two other studies on this subject involving Dutch. Specifically, de Pijper and Sanderman (1994) found that Dutch-speaking listeners associated major prosodic breaks with pauses more strongly than pitch cues, suggesting a more dominant role for pauses. Cambier-Langeveld (2000) found that Dutch-speaking listeners could distinguish between IP boundaries on the one hand and lower-level prosodic boundaries on the other hand, based solely on the degree of final lengthening preceding the boundary, without pause. This finding shows that, at least to some extent, final lengthening is also involved in the identification of prosodic boundaries in Dutch. Yet, since neither study included all cues in the experimental design, it remains unclear how each cue is weighted in Dutch-speaking adults’ perception of IP boundaries. In the current study, we addressed this question by examining the online processing of prosodic boundaries marked by different prosodic cues in Dutch, providing much-needed empirical evidence to support or challenge the claimed reliance on pauses.

Online processing of major prosodic boundaries has been widely studied using electro-encephalography (EEG), and has been associated with a specific event-related potential (ERP) component, the *Closure Positive Shift* (CPS, Steinhauer et al., 1999). The CPS is typically a positive, bilateral neural response to processing major prosodic breaks, occurring between 500 and 800 ms after the onset of the preboundary syllable, although its distribution and latency are affected by utilised stimuli and time-locking points (for a review, see Bögels et al., 2011). It also occurs in response to perceiving prosodic boundaries without explicit attention to the presented stimuli (Peter et al., 2014). By examining the CPS, Holzgrefe-Lang et al. (2016) investigated cue-weighting during IP boundary processing in German-speaking adults. Participants were presented with ambiguous coordinated name sequences, such as *[Moni und Lilli und Manu]* vs. *[Moni und Lilli][und Manu]*. The name sequences were acoustically modified to cue the IP boundary after the second name using either final lengthening, pitch change, or both, without a pause. A CPS response occurred only when both cues were present, but not with either cue alone. Interestingly, these results converged with findings from offline behavioural measures, suggesting that while German-speaking listeners require a combination of pitch and durational cues to process IP boundaries, the pause cue is not essential and has relatively little weight in German.

In the present study, adapting Holzgrefe-Lang et al.’s (2016) methodology, we examined cue-weighting during online processing of utterance-medial IP boundaries in name sequences by native speakers of Dutch. Based on de Pijper and Sanderman (1994), we hypothesise that, in contrast to German, pauses are more important for processing IP boundaries than pitch change or final lengthening in Dutch. Therefore, we expect a weaker CPS (i.e., a lower ERP amplitude) at an IP boundary missing the pause cue, compared to one marked by all three cues, reflecting reduced accuracy in IP boundary processing. However, we predict that CPS amplitude will remain unchanged at an IP boundary missing either final lengthening or pitch change.

## Method

### Participants

Twenty-five native speakers of Dutch (15 female, mean age = 25 years, SD = 5.2 years) were included in the current study. Five additional participants were tested but were excluded from further analysis due to technical problems during testing (n = 2) or having had excessive data loss after preprocessing their EEG data (n = 3). The inclusion criteria included having had their primary and secondary education in areas in which Standard Dutch is spoken, right-handedness, normal or corrected-to-normal vision, and not having had any neurological, psychiatric, hearing or language impairments.

### Stimuli

The stimuli were derived from recordings of a name sequence connected by the coordinating conjunction *en* (‘and’) (see (3) and (4)). A 26-year-old female native speaker of Dutch was instructed to record the name sequence multiple times in a fluent manner as a response to the question *Wie komt daar aan?* (“Who is coming?”). At each rendition, the speaker produced the sequence as either one unit (IP) or as two units (IPs) with a boundary after the second name.

(3) [Moni en Lilli en Manu]_IP_
(4) [Moni en Lilli]_IP_[en Manu]_IP_

Recordings of the renditions were made using a Sennheiser ME-64 microphone at 16 bit/48 kHz in a sound-attenuated shielded cabin at the Utrecht University Institute for Language Sciences Labs. To assess whether the IP boundary in the name sequence was produced accurately, the experimenter (RG) and a second independent rater, who were both naïve as to which sentence was produced, judged the presence or absence of an IP boundary in each recording. They made a note of whether there was a boundary and, if it was present, its location. If the assessments of the two raters were consistent, the recording was retained for further use.

From the retained recordings, six renditions without an IP boundary and six renditions with an IP boundary were subsequently selected based on their naturalness and fluency. These renditions, henceforth referred to as tokens, underwent acoustic analysis to examine whether and how this particular speaker produced prosodic boundaries naturally. Using Praat (Boersma & Weenink, 2022) and ProsodyPro (Xu, 2013), the speaker’s pitch rise in the preboundary syllable, lengthening of the preboundary vowel, and length of pause at the boundary were examined. Tokens with an IP boundary, on average, contained a pitch rise of 118 Hz (6.56 ST) in the preboundary syllable. Compared to tokens without an IP boundary, the duration of the final preboundary vowel was 1.52 times longer. IP boundaries were marked by pauses with an average duration of 894 ms. See Appendix A (Electronic Supplementary Material (ESM)) for detailed results of the acoustic analysis.

These prosodic characteristics were subsequently used to create the experimental stimuli from the six tokens without an IP boundary, ensuring that conditions were identical apart from the manipulation. In total, five conditions were created (see Table 1). The first two conditions contained either no IP boundary (A or the no-boundary condition) or a boundary marked by all three cues (B or the all-cues condition). In the remaining three conditions (C–E), the boundary was signalled by two cues, with each condition omitting a different cue.

**Table 1:** Overview of stimulus conditions and included prosodic cues within each condition

Condition	Description	Included prosodic cues
*A*	no-boundary condition	No cues
*B*	all-cues condition	3 cues: pitch rise, final lengthening and pause
*C*	no-pause condition	2 cues: pitch rise and final lengthening
*D*	no-final-lengthening condition	2 cues: pitch rise and pause
*E*	no-pitch-rise condition	2 cues: final lengthening and pause

Each condition included the same six tokens, with modifications depending on the specific condition. Each token was repeated ten times per condition, yielding 60 trials for each condition. This resulted in a total of 300 stimuli (6 tokens × 5 conditions × 10 repetitions). These 300 stimuli were pseudorandomised into two lists, such that stimuli created from the same token were not presented consecutively, and no more than two stimuli from the same condition were presented consecutively. The two lists were then reversed, creating a total of four experimental lists, which were counterbalanced across participants.

**Fig. 1:**
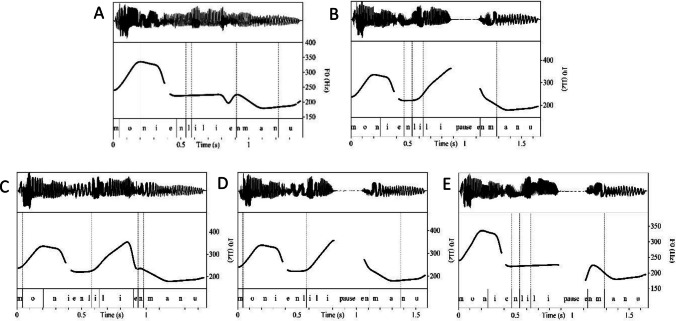
Examples of waveforms (upper part of each illustration), pitch contours (middle), and annotation (lower) of stimuli. **A:** No-boundary condition. **B:** All-cues condition. **C:** No-pause condition. **D:** No-final-lengthening condition. **E:** No-pitch-rise condition

The stimuli were created as follows, using custom-written Praat (Boersma & Weenink, 2022) scripts. First, the pitch contour of every token was simplified to ensure that all conditions, including those in the no-boundary condition, were manipulated to some degree. To simplify the pitch contour, it was stylised to remove pitch points that were under two semitones above or below neighbouring points. These tokens were used in the no-boundary condition (Fig. 1A) and formed the basis for further manipulations to create the stimuli in the four boundary conditions (B–E).

Second, we implemented a pitch rise in *Lilli* in conditions B, C and D (Fig. 1B, C and D, respectively). This was done by measuring the mean pitch of each phoneme in* Lilli* and the mean maximum pitch at the end of *Lilli* in the boundary recordings and changing the pitch contour of the stylized tokens accordingly. This led to a pitch rise of 154 Hz (9.15 semitones), starting at 221 Hz at the beginning of *Lilli* and ending at 375 Hz at the end of *Lilli*.

Third, we implemented the lengthening of the preboundary vowel in conditions B, C and E (Fig. 1B, C and E, respectively) by lengthening the final vowel of *Lilli* by 1.52 times.

Lastly, to implement a pause after *Lilli*, a pause was segmented from a boundary recording and implemented between *Lilli* and *en Manu* in the conditions C, D and E (Fig. 1C, D and E, respectively). Although we aimed to use the prosodic characteristics of the naturally produced boundaries to recreate the individual prosodic cues, this was not feasible for the pause condition. As stated earlier, our acoustic analysis showed that the speaker’s pauses averaged 894 ms. However, informal ratings from native speakers of Dutch not associated with the current study suggested that pauses of 300 ms were the most adequate after the second name in the boundary condition, compared to pauses of 200 ms, 400 ms, 500 ms and 600 ms. Consequently, we chose to use a 300-ms pause in our manipulation. To make the transition from speech to the pause more natural, 100 ms of the end of *Lilli* was used to fade out and 120 ms of the beginning of *en Manu* to fade in. The pause was then implemented by overlapping 50 ms in total on both sides of the pause with speech. This procedure resulted in a pause with a silent interval of 250 ms.

### Procedure

Since the focus of our study was on the automatic processing of prosodic structure, a passive listening paradigm was adopted to elicit the CPS, following Peter et al. (2014). The participants were tested individually in an unshielded EEG laboratory at the Utrecht University Institute for Language Sciences Labs. They sat in front of a monitor and watched a silent nature documentary during the experiment (Forthergill & Linfield, 2007). The participants were instructed to watch the movie whilst listening to the sound that was being played.^1^ To reduce the number of movement artifacts, they were also instructed to blink and move as little as possible. Asynchronously to the movie, the auditory stimulation was delivered via loudspeakers on both sides of the monitor. The trials were presented using Presentation software (Neurobehavioral Systems, 2021), separated by an interstimulus interval of 1,000 ms. The experiment lasted for approximately 15 min, including a short self-paced break every 5 min. The total duration of the participants’ visit was 1 h, including preparation and instruction.

### EEG recording and preprocessing

EEG was continuously recorded at a sampling rate of 2,048 Hz using Actiview software (BioSemi, Brain Products). A standard BioSemi cap with 64 active Ag/AgCl electrodes was used. Additionally, activity on both mastoids, the temples next to each eye, and above and below the left eye was recorded for preprocessing purposes. Impedances of all electrodes were kept below 25 kΩ.

The EEG signal was preprocessed using BrainVision Analyzer (Brain Products GmbH). First, the signal was re-referenced offline to the average activity of the two mastoid electrodes. Second, it was filtered using an IIR zero phrase shift Butterworth bandpass filter (order 2). To remove slow-wave artifacts, for example, caused by breathing, and muscle activation artifacts, frequencies below 0.3 Hz and above 50 Hz were filtered out; to remove line noise, a 50-Hz Notch filter was used. Third, the signal was segmented into epochs which started at 200 ms before the acoustic onset of the final syllable of *Lilli* until 1,100 ms after. Fourth, these epochs were baseline-corrected by subtracting the average activity in the 200 ms prior to the onset of the final syllable of *Lilli* from the rest of the epoch. Fifth and lastly, the epochs were subjected to a two-step automatic artifact rejection, following the same rejection criteria, i.e., maximum allowed voltage step of 50 µV, maximum allowed difference in 100-ms intervals of 200 µV, minimum/maximum allowed amplitude of ± 200 µV, and lowest allowed activity in 100-ms intervals of 0.5 µV. In the first step, trials contaminated with eye movements were removed. To perform this step, two additional channels were created using the signal of the electrodes surrounding the eyes. The signals from the channels next to both eyes were consolidated into one channel showing horizontal eye movements (hEOG); the channels above and below the left eye were used to create a channel reflecting vertical eye movements (vEOG). To identify saccades and blinks, the rejection criteria were applied to the hEOG and vEOG channels, respectively. Epochs in which eye movements were identified were removed across all 64 scalp channels. In the second step, the rejection criteria were applied to each of the 64 scalp channels individually. If an artifact was identified for a particular channel, the epoch was removed only for the respective channel to avoid excessive data loss. Participants were excluded if they had more than 50% trial loss in any of the five conditions (A–E).

After preprocessing, the remaining datasets retained on average 83% of trials in condition A (*M* = 50, *SD* = 8, range = 30–58), 83% in condition B (*M* = 50, *SD* = 9, range = 30–60), 81% in condition C (*M* = 49, *SD* = 8, range = 34–58), 83% in condition D (*M* = 50, *SD* = 8, range = 34–60), and 84% in condition E (*M* = 51, *SD* = 7, range = 32–59).

For statistical analysis, the amplitude in µV averaged over a predefined time window, 500–800 ms after onset of the preboundary syllable (Holzgrefe-Lang et al., 2018), was extracted per channel per participant. Previous research measuring the Closure Positive Shift component using comparable (German) stimuli found effects broadly distributed across the scalp, with the most pronounced results in anterior regions (Holzgrefe-Lang et al., 2016, 2018). In order to investigate this frontal nature of the component in combination with potential hemispheric differences, the electrodes were divided into four regions: Left frontal (henceforth LF: Fp1, AF7, AF3, F7, F5, F3, F1, FT7, FC5, FC3, FC1), right frontal (RF: Fp2, AF8, AF4, F8, F6, F4, F2, FT8, FC6, FC4, FC2), left posterior (LP: TP7, CP5, CP3, CP1, P7, P5, P3, P1, PO7, PO3, O1), and right posterior (RP: TP8, CP6, CP4,CP2, P8, P6, P4, P2, PO8, PO4, O2). Within each region, the electrodes were pooled to improve the signal-to-noise ratio.

### Statistical analysis

Mixed-effect linear modelling was performed with the outcome variable *mean amplitude ERP*, the random factor *participant* and the fixed factor *condition (A, B, C, D, E)* in R Studio (RStudio Team, 2021) with the packages lme4 (Bates et al., 2015) and lmerTest (Kuznetsova et al., 2017). Although the effect of *condition* could vary across scalp regions, we did not formulate specific hypotheses regarding its spatial distribution. To account for the possibility that effects in the opposite direction across regions (e.g., a positive effect in the left hemisphere and a negative effect in the right) might cancel each other out when averaged, we examined the effect of the factor *condition* within each region separately. The factor *condition* was thus nested across *region* (LF, RF, LP, RP). The maximal model was first constructed, and in the case of non-convergence, its random structure was simplified to a reduced model (see Barr et al., 2013). Thus, the random structure of the maximal model included a random slope for *condition* nested across *region* by *participant*,^2^ resulting in *(region/condition + (region/condition | participant).* However, this model was unable to converge. The random structure was therefore simplified to include only a random slope for *condition*. This model, *(region/condition + (condition | participant)*, was able to converge and its results are reported in the *Results* section.

To test our predictions, four comparisons were created by coding *condition*. In the first comparison, we examined whether the boundary had an impact on processing. In this case, the no-boundary condition was compared with the average of the four boundary conditions. Therefore, coding was done using sum-to-zero contrasts, resulting in condition A coded as −1 and conditions B, C, D and E each as one-quarter. The second, third and fourth comparison examined whether leaving out a certain cue affected boundary perception. In each of these comparisons, we compared the all-cues condition (condition B) with each of the boundary conditions lacking one cue (conditions C, D or E). This approach can reveal what cues are essential in the processing of boundaries. If the omission of a cue results in a reduced amplitude of the CPS or absence of the CPS, it means that the unavailable cue plays a role (in the former scenario) or is required (in the latter scenario) to process the IP boundary. Conversely, if the omission of a cue leads to no significant differences in the amplitude of the CPS, it means the cue contributes little to online processing of IP boundaries in the presence of the other cues. To implement the second, third and fourth comparisons, condition B (all cues) was coded as −1 and conditions C, D or E were coded as 1. All the comparisons were implemented into a coding matrix, which was then transformed using its generalised inverse to create a contrast matrix for linear modelling, following Schad et al. (2020).

## Results

### Effect of boundary

**Fig. 2:**
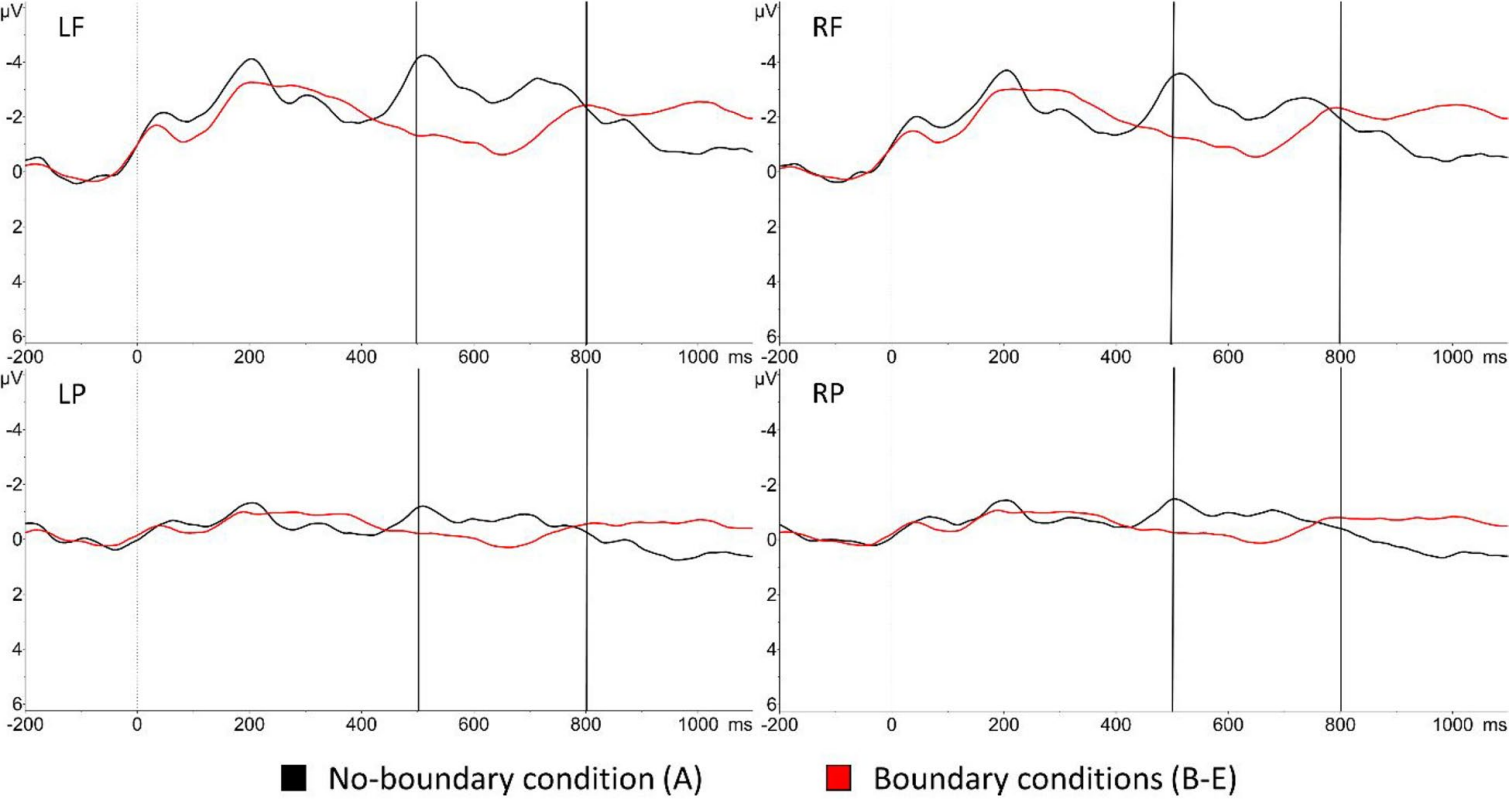
Event-related potential (ERP) waveforms for no-boundary-condition vs. averaged boundary conditions. ERP waveforms are low-pass filtered at 8 Hz for illustration purposes. Upper left: left frontal (**LF**); upper right: right frontal (**RF**); lower left: left posterior (**LP**); lower right: right posterior (**RP**). Vertical dotted line: onset of the preboundary syllable. Vertical solid lines correspond to the analysed time-window (500–800 ms)

Compared to the no-boundary condition (A), the average amplitude of the boundary conditions (B–E) was significantly more positive in all regions (LF: β = 1.60, *SD* = 0.18*, t* = 8.715,* p* < 0.001; RF: β = 1.35, *SD* = 0.18, *t* = 7.354, *p* < 0.001; LP: β = 0.67, *SD* = 0.18, *t* = 3.632, *p* < 0.001; RP: β = 0.70, *SD* = 0.18, *t* = 3.779, *p* < 0.001). Figure 2 presents ERP waveforms for the no-boundary condition and the combined boundary conditions, demonstrating a more positive deflection for the latter in the specified time-window.

### Effect of leaving out pause

**Fig. 3:**
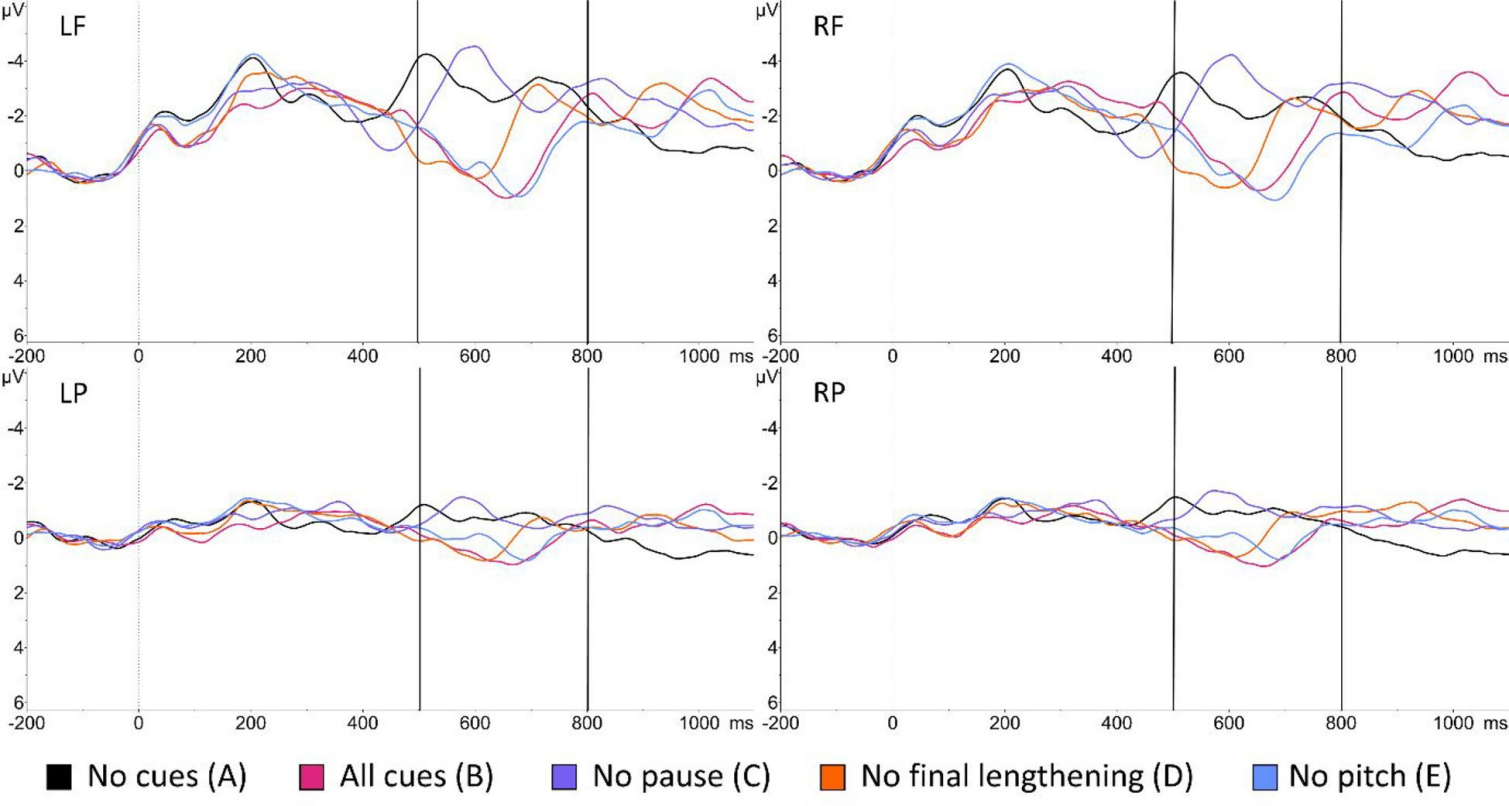
Event-related potential (ERP) waveforms in four regions, time-locked to preboundary syllable onset. ERP waveforms are low-pass filtered at 8 Hz for illustration purposes. Upper left: left frontal; upper right: right frontal; lower left: left posterior; lower right: right posterior. Vertical dotted line: onset of the preboundary syllable. Vertical solid lines correspond to the analysed time-window (500–800 ms)

In the absence of the pause cue (C), the amplitude decreased significantly across all regions compared to the all-cues condition (B) (LF: β = −2.32, *SD* = 0.36, *t* = −6.424, *p* < 0.001; RF: β = −2.36, *SD* = 0.36, *t* = −6.523, *p* < 0.001; LP: β = −1.18, *SD* = 0.36*, t* = −3.266, *p* = 0.003; RP: β = −1.50, *SD* = 0.36, *t* = −4.163, *p* < 0.001). The ERP waveforms of the no-pause condition and the all-cues condition differed substantially across all regions (Fig. 3). Instead, the no-pause condition elicited an ERP resembling the waveform shape of the no-boundary condition (A), although the amplitude peak shifted in time due to final lengthening.

### Effect of leaving out final lengthening

When comparing the all-cues condition (B) with the no-final-lengthening condition (D), the amplitude did not differ significantly (*p* = 0.13, 0.68, 0.53 and 0.30 in LF, RF, LP and RP region, respectively). As shown in Fig. 3, the waveform for the no-final-lengthening condition resembled the shape and amplitude of the all-cues condition. The amplitude peak occurred earlier though, due to the shorter duration of the preboundary syllable in the no-final-lengthening condition.

### Effect of leaving out pitch rise

Similarly, we did not find any significant amplitude differences between the all-cues condition (B) and the no-pitch-rise condition (E) (*p* = 0.93, 0.20, 0.48 and 0.40 in LF, RF, LP and RP region, respectively). The waveforms between the no-pitch-rise condition and the all-cues condition were also remarkably similar (Fig. 3).

In summary, we found a positive shift in the window of interest (i.e., 500–800 ms after the onset of the preboundary syllable) in the average ERPs of the four boundary conditions (B–E). Furthermore, ERP amplitude decreased significantly when the boundary lacked a pause cue (C) compared to when all three cues were present (B). However, removing final lengthening or pitch rise from IP boundaries did not affect ERP amplitude.

Subsequent exploratory analysis (Appendix B, ESM) revealed that ERP amplitude was significantly higher in the all-cue, no-final-lengthening, and no-pitch-rise conditions compared to the no-boundary condition. A second exploratory analysis (Appendix C, ESM) examined whether the positive shift in the all-cues, no-final-lengthening, and no-pitch-rise conditions was a genuine CPS, following Männel (2009), and confirmed that it was the CPS. Thus, the CPS was present in all boundary conditions except for the no-pause condition. A third exploratory analysis (Appendix D, ESM) assessed potential latency effects from final lengthening, and confirmed that the latency differences across conditions did not affect our main analysis results.

## Discussion

Using the ERP paradigm, the current study aimed to investigate cue-weighting in processing utterance-medial IP boundaries in Dutch. The participants passively listened to sequences of coordinated names, either without or with a prosodic boundary marked by varying combinations of cues. Compared to stimuli without an IP boundary, stimuli with an IP boundary elicited a global CPS, indicating that participants perceived and processed the prosodic boundary. Our analysis shows that pause is a key cue for IP boundary processing in Dutch-speaking listeners. CPS appeared in conditions with a pause (B, D, E) and was absent in those without it (A, C), suggesting that the pause cue is required for native speakers of Dutch to process IP boundaries in syntactically ambiguous utterances. Leaving out final lengthening or pitch rise did not alter the CPS amplitude, suggesting neither cue is necessary for boundary processing when two other cues, including pause, are available. These results are consistent with our hypothesis based on previous research in Dutch, showing that native speakers of Dutch more strongly associate major prosodic boundaries with pauses than pitch changes (de Pijper & Sanderman, 1994).

Notably, the present findings on adult Dutch speakers’ boundary perception align with behavioural evidence in Dutch-learning infants. Like Dutch-speaking adults, Dutch-learning 6-month-olds rely on the pauses to recognize (sentence-final) IP boundaries, which differs from their English-acquiring and German-acquiring peers who rely on pitch-related and final lengthening cues (Holzgrefe-Lang et al., 2018; Johnson & Seidl, 2008; Seidl, 2007; Seidl & Cristià, 2008; Wellmann et al., 2012). This suggests that 6-month-old infants may have acquired language-specific cue-weighting in the perception of IP boundaries in Dutch, English and German. However, these infant studies rely on the offline ability to detect acoustic anomalies in the transition from one sentence to another, whereas the current EEG study and Holzgrefe-Lang et al. (2018) test online processing of prosodic cues at IP transitions within the same utterance, an albeit more stringent perceptual task. Research examining IP boundary processing using EEG in 6-month-old infants is needed to examine when infants acquire adult-like cue-weighting in online processing of prosodic boundaries in Dutch and other languages.

On the crosslinguistic level, we found both similarities and differences in the processing of IP boundaries by native speakers of Dutch and German. The CPS responses found in the current study are highly similar to those observed in German-speaking listeners in terms of localisation: it is a broadly distributed, bilateral response that is most evident in the frontal regions (Holzgrefe et al., 2013; Holzgrefe-Lang et al., 2016, 2018). This similarity suggests that the processing mechanisms underlying the CPS are the same across speakers of both languages, regardless of cue-weighting differences.

However, the observed heavy weighting of the pause cue in the current study contrasts with previous findings on German speakers. With methods and stimuli closely resembling those employed in the present study, the CPS is found in German-speaking listeners presented with IP boundaries marked by pitch change and final lengthening (Holzgrefe-Lang et al., 2016, 2018). This demonstrates that the pause is a non-essential cue in German.

If the processing mechanisms are indeed identical, then the question arises as to why Dutch differs from German and English in the weighting of prosodic boundary cues, even though these West-Germanic languages are traditionally considered prosodically similar (see, e.g., Dauer, 1983; Langus et al., 2017; Nespor et al., 2011). A possible explanation could be that the relative weighting in perception reflects the language-specific realisation of cues in production. For instance, a corpus study of spontaneous speech in German found that pauses are produced less frequently as prosodic boundary markers than final lengthening and pitch change, and are thus considered optional (Peters et al., 2005). This could lead to a lower weighting of pauses during boundary perception.

Regarding Dutch, it has been observed that across pitch events, native speakers of Dutch use smaller pitch spans than native speakers of British English (de Pijper, 1983; Willems, 1982). Native speakers of German also use smaller pitch spans than native speakers of British English (Gibbon, 1998) and American English (Jilka, 2000), but arguably still larger pitch spans than Dutch, at least at IP boundaries. Some evidence for this difference between Dutch and German comes from Holzgrefe-Lang et al. (2016). Within the same name sequences, the German IP boundary had a pitch rise of 206 Hz (13.16 semitones), while this study used a rise of 154 Hz (9.15 semitones). In both cases, the adopted values were selected based on typical recordings of native speakers, suggesting more acoustically salient pitch changes in German. Boundary production in American English also involves a larger pitch rise in the preboundary syllable compared to Dutch (Zhang, 2012). Similar differences in cue realisation between English, German and Dutch apply to final lengthening. Increase in syllabic duration is larger in English than in Dutch, since lengthening can arise from both accentuation and the IP-final position in English, while only the preboundary position leads to lengthening in Dutch (Cambier-Langeveld, 2000). Possibly, final lengthening is more extensive in German than in Dutch too. Evidence for this difference comes from comparing stimuli in Holzgrefe-Lang et al. (2016) and this study, where the same preboundary vowel was lengthened by a factor of 1.8 in German versus 1.52 in Dutch. In sum, reduced pitch and lengthening cues in spoken Dutch may make them less salient, leading to a greater reliance on pause. Crosslinguistic differences in the acoustic realisation of prosodic boundary cues may thus contribute to language-specific cue-weighting in general, and specifically to the observed contrast between Dutch and German.^3^

Importantly, differences in experimental setup may also have contributed to the observed contrast between Dutch and German. In the current experimental paradigm, the participants listened passively to the presented stimuli while watching a non-related video, whereas other experiments involved active listening by using an additional forced-choice task (Holzgrefe-Lang et al., 2016). Although there have been no studies on the influence of both experimental approaches on the CPS component, it has been suggested that passive CPS differs from active CPS in terms of topography, latency and amplitude (Peter et al., 2014). Still, it is unclear whether active listening in an attention-demanding context can enhance the relative weighting of individual prosodic cues, leading to processing differences relative to passive listening. Future research is needed to shed light on the interaction between attention and cue-weighting. In addition, to gain more insight into potential crosslinguistic differences, future work should directly compare multiple languages within a unified experimental framework.

Finally, the generalisability of our results needs validation. Specifically, the stimuli were globally ambiguous, derived from recordings without an IP boundary, with disambiguation relying solely on local prosodic cues. However, listeners may use other available cues to perceive prosodic boundaries. For example, in similar coordinated name sequences non-local cues may also influence perception, including prosodic phrase-initial weakening, i.e., reduced duration and pitch range on the first of two coordinated elements (Hansen et al., 2023; Kentner & Féry, 2013; Wagner, 2005) and segmental phrase-initial strengthening (Cho, 2016). Identifying prosodic boundaries involves a complex cognitive integration of various types of (non-)linguistic information, including prosodic cues, phonetic markers, lexico-syntactic knowledge, discourse considerations, utterance length, and visual cues (Cho, 2016; Clifton et al., 2006; Esteve-Gibert & Prieto, 2013; Itzhak et al., 2010; Kerkhofs et al., 2007). It is therefore unclear whether the same patterns of cue-weighting, particularly the importance of pauses, can be observed across different speech materials, contexts or speaker populations. In particular, listener-specific factors such as second-language experience and musical training have been suggested to contribute to individual differences in prosodic boundary perception among speakers of the same language (van Ommen et al., 2020). Further work is needed to validate the generalisability of the current results using stimuli more representative of the distribution of boundary-marking cues in naturally occurring speech and across speakers with diverse linguistic backgrounds. Moreover, future research could explore how cue-weighting in prosodic boundary processing affects early language acquisition, and potentially contribute to the early detection of atypical language development.

## Conclusion

This study examined processing of IP boundaries in coordinated constructions in Dutch, exploring the role of individual prosodic cues using EEG. We presented native speakers of Dutch with spoken coordinated name sequences with an intervening IP boundary marked by different cue combinations. We found a CPS when the boundaries were marked by pitch change, final lengthening, and pause, or by a pause and either final lengthening or pitch change. When pauses were absent, no CPS was obtained, suggesting that pauses form a highly important cue for IP boundary perception and processing in Dutch. These results contrast with previous findings in German, indicating that the observed reliance on the pause cue reflects language-specific cue-weighting. Future research using a similar methodology to investigate online processing of prosodic boundaries in other languages like English is needed to better understand crosslinguistic similarities and differences.

## Supplementary Information

Below is the link to the electronic supplementary material.

Supplementary file1 (DOCX 663 KB)

## Data Availability

Data, materials and analysis code are available at 10.24416/UU01-L84ZFK.

## Footnotes

1 Although processing of the visual input from the movie contributed to overall brain activity, the video was presented asynchronously to the auditory stimuli. As a result, visually evoked responses were not time-locked to the auditory events of interest and therefore did not systematically contribute to the averaged ERPs, cancelling out across trials due to their random timing. Any constant background effects of watching a movie would have been present across all conditions and thus cannot account for the condition-specific ERP differences observed.

2 We did not include a random effect of the experimental list that a participant was exposed to as there was too little variation across participants. Furthermore, the potential effect of a particular list is mitigated by pseudorandomisation and using the reversed counterparts of lists.

3 A valid comparison with cue-weighting in prosodic boundary perception in English cannot be made, as prior studies on boundary cue weighting in English did not focus on online processing (Mo, 2010; Zhang, 2012). Additionally, these studies did not distinguish different levels of prosodic boundaries (Mo, 2010), and the pitch manipulation employed was not comparable to production data (Zhang, 2012, p. 60).

## References

[CR1] Barr, D. J., Levy, R., Scheepers, C., & Tily, H. J. (2013). Random effects structure for confirmatory hypothesis testing: Keep it maximal. *Journal of Memory and Language,**68*(3), 255–278. 10.1016/j.jml.2012.11.001 10.1016/j.jml.2012.11.001 PMC3881361 24403724

[CR2] Bates, D., Mächler, M., Bolker, B. M., & Walker, S. C. (2015). Fitting linear mixed-effects models using lme4. *Journal of Statistical Software*, *67*(1). 10.18637/jss.v067.i01

[CR3] Beckman, M. E., & Pierrehumbert, J. B. (1986). Intonational structure in Japanese and English. *Phonology Yearbook,**3*, 255–309.

[CR4] Boersma, P., & Weenink, D. (2022).* Praat: doing phonetics by computer* [Software]. Version 6.2.17. Retrieved August 23, 2022 from http://www.praat.org/

[CR5] Bögels, S., Schriefers, H., Vonk, W., & Chwilla, D. J. (2011). Prosodic breaks in sentence processing investigated by event-related potentials. *Language and Linguistics Compass,**5*(7), 424–440. 10.1111/j.1749-818X.2011.00291.x

[CR6] Brain Products GmbH. (2021). *BrainVision Analyzer (v 222)*. Gilching.

[CR7] Cambier-Langeveld, G. M. (2000). *Temporal Marking of Accents and Boundaries [Doctoral dissertation]*. University of Amsterdam.

[CR8] Chen, A. (2007). Language-specificity in the perception of continuation intonation. *Tones and tunes II: Phonetic and behavioural studies in word and sentence prosody* (pp. 107–142). Mouton de Gruyter. 10.1515/9783110207576.1.107

[CR9] Cho, T. (2016). Prosodic boundary strengthening in the phonetics-prosody interface. *Language and Linguistics Compass,**10*(3), 120–141. 10.1111/lnc3.12178

[CR10] Clifton, C., Carlson, K., & Frazier, L. (2006). Tracking the what and why of speakers’ choices prosodic boundaries and the length of constituents. *Psychonomic Bulletin & Review,**13*(5), 854–861. 17328385 10.3758/bf03194009

[CR11] Dauer, R. M. (1983). Stress-timing and syllable-timing reanalyzed. *Journal of Phonetics,**11*(1), 51–62.

[CR12] de Pijper, J. R. (1983). *Modelling British English Intonation*. De Gruyter. 10.1515/9783110883510

[CR13] de Pijper, J. R., & Sanderman, A. A. (1994). On the perceptual strength of prosodic boundaries and its relation to suprasegmental cues. *Journal of the Acoustical Society of America,**96*(4), 2037–2047. 10.1121/1.410145

[CR14] Esteve-Gibert, N., & Prieto, P. (2013). Prosodic structure shapes the temporal realization of intonation and manual gesture movements. *Journal of Speech, Language, and Hearing Research,**56*(3), 850–864. 10.1044/1092-4388(2012/12-0049) 23275426 10.1044/1092-4388(2012/12-0049)

[CR15] Ferreira, F. (1993). Creation of prosody during sentence production. *Psychological Review,**100*(2), 233–253. 8483983 10.1037/0033-295x.100.2.233

[CR16] Gibbon, D. (1998). Intonation in German. In D. Hirst & A. di Cristo (Eds.), *Intonation systems: A survey of twenty languages* (8th ed., pp. 78–95). University Press.

[CR17] Forthergill, A., & Linfield, M. (2007). *Planet Earth, Seasonal Forests (Season 1, Episode 10) [Broadcast]*. BBC One.

[CR18] Grice, M. (2006). Intonation. In K. Brown (Ed.), *Encyclopedia of Language and Linguistics* (2nd ed., Vol. vol. 5, pp. 778–788). Elsevier.

[CR19] Gussenhoven, C. (2004). *The Phonology of Tone and Intonation*. Cambridge University Press.

[CR20] Hansen, M., Huttenlauch, C., de Beer, C., Wartenburger, I., & Hanne, S. (2023). Individual differences in early disambiguation of prosodic grouping. *Language and Speech,**66*(3), 706–733. 36250333 10.1177/00238309221127374

[CR21] Holzgrefe, J., Wellmann, C., Petrone, C., Truckenbrodt, H., Höhle, B., & Wartenburger, I. (2013). Brain response to prosodic boundary cues depends on boundary position. *Frontiers in Psychology,**4*, 421. 23882234 10.3389/fpsyg.2013.00421 PMC3714540

[CR22] Holzgrefe-Lang, J., Wellmann, C., Petrone, C., Räling, R., Truckenbrodt, H., Höhle, B., & Wartenburger, I. (2016). How pitch change and final lengthening cue boundary perception in German: Converging evidence from ERPs and prosodic judgements. *Language, Cognition and Neuroscience,**31*(7), 904–920. 10.1080/23273798.2016.1157195

[CR23] Holzgrefe-Lang, J., Wellmann, C., Höhle, B., & Wartenburger, I. (2018). Infants’ processing of prosodic cues: Electrophysiological evidence for boundary perception beyond pause detection. *Language and Speech,**61*(1), 153–169. 10.1177/0023830917730590 28937300 10.1177/0023830917730590

[CR24] Horne, M., Strangert, E., & Heldner, M. (1995). Prosodic boundary strength in Swedish: Final lengthening and silent interval duration. *Proceedings of the 13th International Congress of Phonetic Sciences,**1*, 170–173.

[CR25] Houston, D. M., Jusczyk, P. W., Kuijpers, C., Coolen, R., & Cutler, A. (2000). Cross-language word segmentation by 9-month-olds. *Psychonomic Bulletin & Review,**7*(3), 504–509. 11082857 10.3758/bf03214363

[CR26] Itzhak, I., Pauker, E., Drury, J. E., Baum, S. R., & Steinhauer, K. (2010). Event-related potentials show online influence of lexical biases on prosodic processing. *Neuroreport,**21*(1), 8–13. 10.1097/WNR.0b013e328330251d 19884867 10.1097/WNR.0b013e328330251d

[CR27] Jilka, M. (2000). *The contribution of intonation to the perception of foreign accent*. University of Stuttgart.

[CR28] Johnson, E. K., & Seidl, A. (2008). Clause segmentation by 6-month-old infants: A crosslinguistic perspective. *Infancy,**13*(5), 440–455. 10.1080/15250000802329321

[CR29] Kentner, G., & Féry, C. (2013). A new approach to prosodic grouping. *The Linguistic Review,**30*(2), 277–311. 10.1515/tlr-2013-0009

[CR30] Kerkhofs, R., Vonk, W., Schriefers, H., & Chwilla, D. J. (2007). Discourse, syntax, and prosody: The brain reveals an immediate interaction. *Journal of Cognitive Neuroscience,**19*(9), 1421–1434. 17714005 10.1162/jocn.2007.19.9.1421

[CR31] Kuijpers, C., Coolen, R., Houston, D., & Cutler, A. (1998). Using the Head-Turning Technique to Explore Cross-Linguistic Performance Differences. In L. P. Lipsitt & C. K. Rovee-Collier (Eds.), *Advances in infancy research* (Vol. 12, pp. 205–220). Ablex.

[CR32] Kuznetsova, A., Brockhoff, P. B., & Christensen, R. H. B. (2017). LmerTest package: Tests in linear mixed effects models. *Journal of Statistical Software,**82*(13), 1–26. 10.18637/JSS.V082.I13

[CR33] Ladd, D. R. (2008). Prosodic structure. *Intonational Phonology* (pp. 281–309). Cambridge University Press. 10.1017/cbo9780511808814.009

[CR34] Langus, A., Mehler, J., & Nespor, M. (2017). Rhythm in language acquisition. *Neuroscience and Biobehavioral Reviews,**81*, 158–166. 10.1016/j.neubiorev.2016.12.012 27993604 10.1016/j.neubiorev.2016.12.012

[CR35] Männel, C. (2009) *Prosodic processing during language acquisition: Electrophysiological studies on Intonational phrase processing* [Doctoral dissertation]. Max Planck Institute for Human Cognitive and Brain Sciences Leipzig

[CR36] Mo, Y. (2010). *Prosody production and perception with conversational speech [Doctoral dissertation]*. University of Illinois Urbana-Champaign.

[CR37] Nespor, M., & Vogel, I. (1986). *Prosodic Phonology*. Foris.

[CR38] Neurobehavioral Systems, Inc. (2021). *Presentation* [Software] (version 23.0, 27 October 2021).

[CR39] Nespor, M., Shukla, M., & Mehler, J. (2011). Stress-timed vs. syllable timed languages. In M. van Oostendorp, C. J. Ewen, E. Hume, & K. Rice (Eds.), *The Blackwell Companion to Phonology* (pp. 1147–1159). Wiley-Blackwell.

[CR40] Peter, V., Mcarthur, G., & Crain, S. (2014). Using event-related potentials to measure phrase boundary perception in English. *BMC Neuroscience,**15*(129), 1–11. 10.1186/s12868-014-0129-z 25424987 10.1186/s12868-014-0129-z PMC4252994

[CR41] Peters, B., Kohler, K. J., & Wesener, T. (2005). Phonetische Merkmale prosodischer Phrasierung in deutscher Spontansprache. In K. J. Kohler, F. Kleber, & B. Peters (Eds.), *AIPUK. Prosodic structures in German spontaneous speech* (Vol. 35, pp. 143–184). IPDS.

[CR42] Redi, L., & Shattuck-Hufnagel, S. (2001). Variation in the realization of glottalization in normal speakers. *Journal of Phonetics,**29*, 407–429. 10.1006/jpho.2001.0145

[CR43] RStudio Team. (2021). *RStudio: Integrated Development Environment for R*. RStudio PBC.

[CR44] Schad, D. J., Vasishth, S., Hohenstein, S., & Kliegl, R. (2020). How to capitalize on a priori contrasts in linear (mixed) models: A tutorial. *Journal of Memory and Language*. 10.1016/j.jml.2019.104038

[CR45] Seidl, A. (2007). Infants’ use and weighting of prosodic cues in clause segmentation. *Journal of Memory and Language,**57*(1), 24–48. 10.1016/j.jml.2006.10.004

[CR46] Seidl, A., & Cristià, A. (2008). Developmental changes in the weighting of prosodic cues. *Developmental Science,**11*(4), 596–606. 10.1111/j.1467-7687.2008.00704.x 18576967 10.1111/j.1467-7687.2008.00704.x

[CR47] Selkirk, E. (2005). Comments on intonational phrasing in English. In S. Frota, M. Vigário, & M. Freitas (Eds.), *Prosodies: With special reference to Iberian languages* (pp. 11–58). Mouton de Gruyter.

[CR48] Steinhauer, K., Alter, K., & Friederici, A. D. (1999). Brain potentials indicate immediate use of prosodic cues in natural speech processing. *Nature Neuroscience,**2*(2), 191–196. 10195205 10.1038/5757

[CR49] van Ommen, S., Boll-Avetisyan, N., Larraza, S., Wellmann, C., Bijeljac-Babic, R., Höhle, B., & Nazzi, T. (2020). Language-specific prosodic acquisition: A comparison of phrase boundary perception by French- and German-learning infants. *Journal of Memory and Language*. 10.1016/j.jml.2020.104108

[CR50] Wagner, M. (2005). *Prosody and Recursion [Doctoral dissertation]*. Massachusetts Institute of Technology.

[CR51] Wagner, M., & Watson, D. G. (2010). Experimental and theoretical advances in prosody: A review. *Language and Cognitive Processes,**25*(7), 905–945. 10.1080/01690961003589492 22096264 10.1080/01690961003589492 PMC3216045

[CR52] Wellmann, C., Holzgrefe, J., Truckenbrodt, H., Wartenburger, I., & Höhle, B. (2012). How each prosodic boundary cue matters: Evidence from German infants. *Frontiers in Psychology*, *3*(DEC). 10.3389/fpsyg.2012.00580 10.3389/fpsyg.2012.00580 PMC3533501 23293618

[CR53] Willems, N. (1982). *English intonation from a Dutch point of view*. Foris.

[CR54] Xu, Y. (2013). ProsodyPro-A Tool for Large-scale Systematic Prosody Analysis. *Proceedings of Tools and Resources for the Analysis of Speech Prosody* (pp. 7–10). Aix-en-Provence.

[CR55] Yang, X., Shen, X., Li, W., & Yang, Y. (2014). How listeners weight acoustic cues to intonational phrase boundaries. *PLoS One*. 10.1371/journal.pone.0102166 25019156 10.1371/journal.pone.0102166 PMC4096911

[CR56] Zhang, X. (2012). *A comparison of cue-weighting in the perception of prosodic phrase boundaries in English and Chinese [Doctoral dissertation]*. University of Michigan.

