# Supplementary material for: Cue-weighting in processing of prosodic boundaries in Dutch: An event-related potential (ERP) study
Source: Psychon Bull Rev. 2026 Jan 20;33(1):53. doi: 10.3758/s13423-025-02843-x (PMC12819481; doi:10.3758/s13423-025-02843-x)
Supplement: Supplementary file 1 — Supplementary file1 (DOCX 663 KB) [file 13423_2025_2843_MOESM1_ESM.docx]

# Appendix A: Results acoustic analysis

Results from the acoustic analysis are represented in Figure A1-A4 and values can be found in Table A1. In Figure A1-A3, red represents tokens without a boundary, and blue represents tokens with a boundary.

**Figure A1**

*Acoustic analysis of mean fundamental frequency of each phoneme in ‘Lilli’*


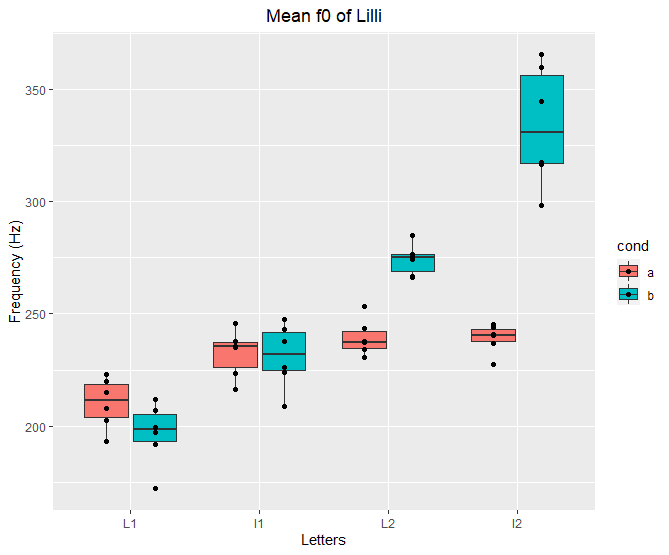


**Figure A2**

*Acoustic analysis of mean duration of each phoneme in ‘Lilli’*


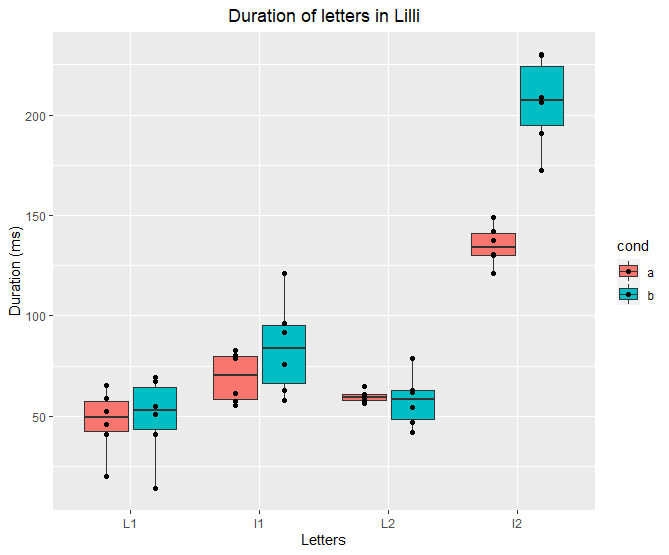


**Figure A3**

*Acoustic analysis of minimum (top), maximum (middle), and rise in fundamental frequency (bottom) of each phoneme in ‘Lilli’*


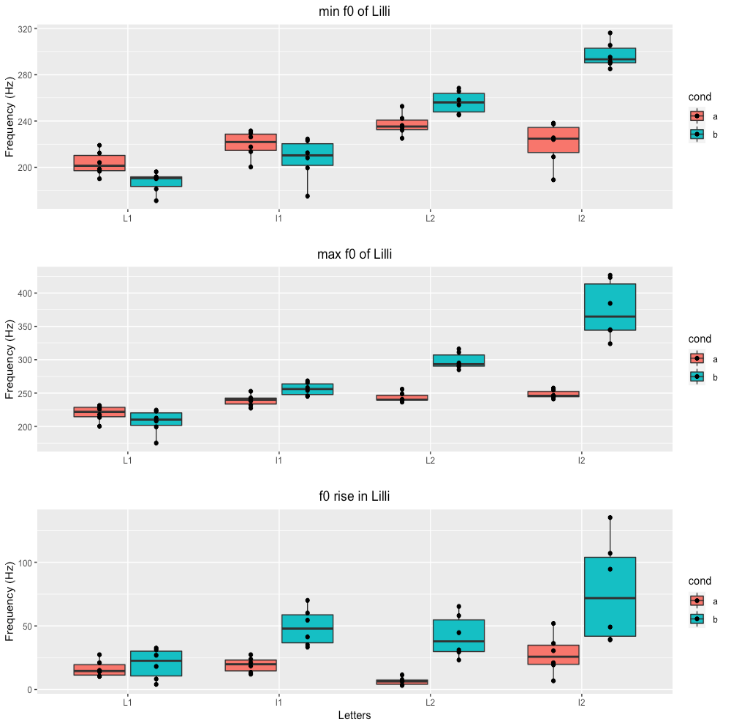


**Figure A4**

*Acoustic analysis of pause duration in tokens with a boundary (condition B).*


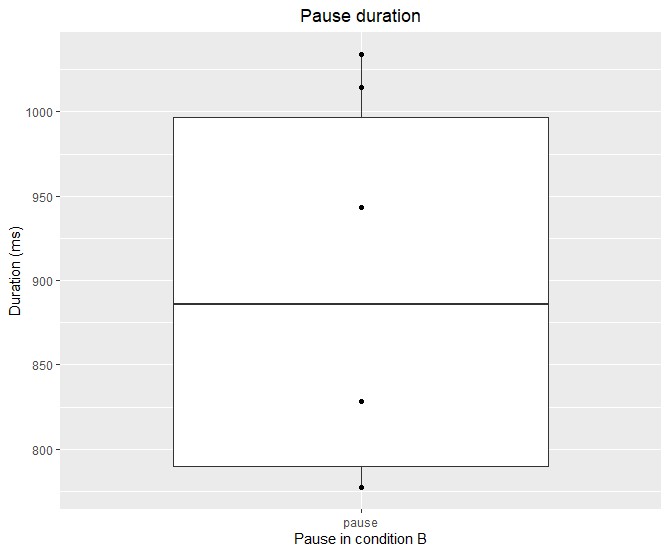


**Table A1**

*Mean values and ranges of pitch rise, maximum pitch, vowel duration in the second syllable of the second name, and pause duration after the second name in sequences with and without an utterance-medial IP boundary*

|  | Pitch rise in Hz  (min.-max.) | Max. pitch in Hz (min.-max.) | Final preboundary vowel duration in ms (min.-max.) | Pause duration in ms (min.-max.) |
| --- | --- | --- | --- | --- |
| *Without boundary* | 11 (225-258) | 248 (241-258) | 135 (121-149) | *NA* |
| *With boundary* | 118 (245-427) | 375 (324-427) | 206 (172-230) | 896 (777-1034) |

# Appendix B: Exploratory analysis of the presence of the CPS in boundary conditions

To examine whether we found a CPS in all four boundary conditions, we compared each boundary condition to the no-boundary condition. Using the same model as reported for the main analysis, the conditions were dummy coded, with the no-boundary condition (A) coded as 0 and each of the boundary conditions coded as 1 in subsequent comparisons.

### Effect of the all-cues boundary

Compared to the no-boundary condition (A), the all-cues condition (B) was more significantly positive across all regions (LF: β = 2.36 µV, *SD* = 0.27, *t* = 8.902, *p* < 0.001; RF: β = 1.87, µV, *SD* = 0.27, *t* = 7.021, *p* < 0.001; LP: β = 1.11 µV, *SD* = 0.27, *t* = 4.176, *p* < 0.001; RP: β = 1.29 µV, *SD* = 0.27, *t* = 4.832, *p* < 0.001).

### Effect of the no-pause boundary

Compared to the no-boundary condition (A), the no-pause condition (C) resulted in significant negativity in the right frontal region (β = -0.49 µV, *SD* = 0.23*, t* = -2.119, *p* = 0.42). This effect was not significant in the other regions (LF: *p* = 0.86; LP: *p* = 0.76; RP: *p* = 0.35). The negativity in the frontal region might be the result of an overall more negative placement of the waveform (Figure 3 in the main text). This can be explained by differences in latency due to the presence of final lengthening.

### Effect of the no-final-lengthening boundary

Compared to the no-boundary condition (A), the no-final-lengthening condition (D) was significant more positive in the frontal regions (LF: β = 1.06 µV, *SD* = 0.44, *t* = -3.662, *p* = 0.001; RF: β = 1.67 µV, *SD* = 0.44, *t* = 3.799, *p* < 0.001). No significant differences were found in the posterior regions (LP: *p* = 0.08; RP: *p* = 0.09), which might be due to latency differences. By removing final lengthening, the small positive peak went back to baseline halfway the time-window.

### Effect of the no-pitch-rise boundary

Compared to the no-boundary condition (A), the no-pitch-rise condition (E) resulted in significant positivity across all regions (LF: β = 2.40 µV, *SD* = 0.27, *t* = 9.010, *p* < 0.001; RF: β = 2.37 µV, *SD* = 0.27, *t* = 3.561, *p* < 0.001; LP: β = 0.83 µV, *SD* = 0.27, *t* = 3.114, *p* < 0.001; RP: β = 0.95 µV, *SD* = 0.27, *t* = 3.561, *p* < 0.001).

The results of this exploratory analysis showed that the responses to the all-cues boundary, the no-final-lengthening boundary, and the no-pitch-rise boundary significantly differed from the responses to the no-boundary condition. However, this was not the case for no-pause boundary, in which the responses did not significantly differ from or were slightly more negative than the no-boundary condition. This suggest that the responses to the no-boundary condition and the no-pause condition were similar, and thus that we did not find a CPS in the no-pause condition.

# ****Appendix C: Exploratory analysis to check if the positivity is the CPS****

This analysis was based on one done by Männel (2009). The author hypothesised that, by comparing the obligatory evoked potentials at the onset of the stimulus and the onset of the second phrase, one can examine whether participants only processed the stimuli acoustically or whether they processed the boundary as well. In the former, the obligatory evoked potentials should be similar in shape, whereas in the latter, there should be an additive effect of the CPS in the waveform. The obligatory potentials we focused on were the N1, a negative peak at around 100 ms after the time-locking point, and P2, a positive peak at around 200 ms.

Figures C1 and C2 were made by time-locking on the onset of the stimulus (C1) and the third name (*en Manu*; C2). The same preprocessing steps as reported in the methods were applied here, except that the epochs for Figure C2 were slightly shorter to retain more trials.

**Figure C1**

*ERP waveforms in four regions, time-locked to stimulus onset*


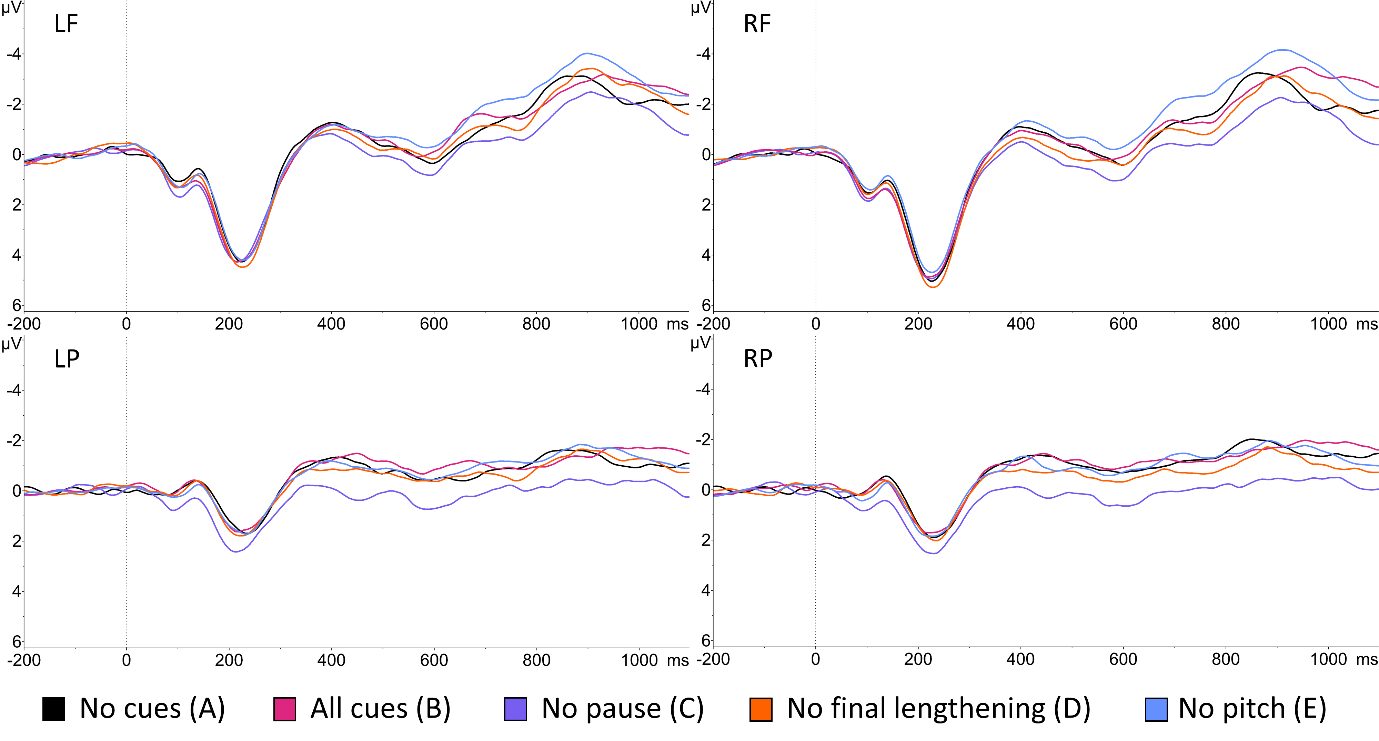


*Note.* ERP waveforms are low-pass filtered at 8Hz for illustration purposes. Upper left: left frontal; upper right: right frontal; lower left: left posterior; lower right: right posterior. Vertical dotted line: onset of the stimulus.

**Figure C2**

*ERP waveforms in four regions, time-locked to second phrase onset*


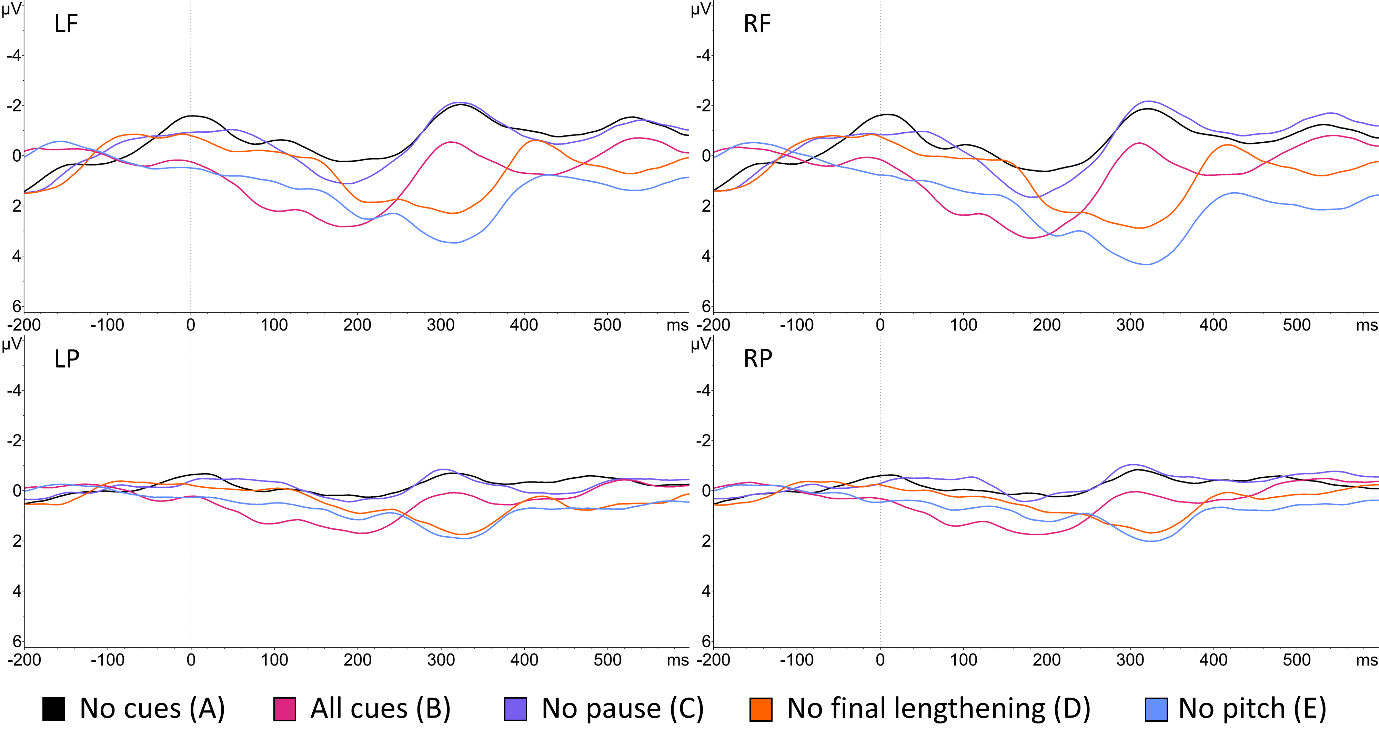


*Note.* ERP waveforms are low-pass filtered at 8Hz for illustration purposes. Upper left: left frontal; upper right: right frontal; lower left: left posterior; lower right: right posterior. Vertical dotted line: onset of the second phrase.

We decided to not perform a statistical analysis as there is a stark difference between stimulus onset and the onset of third name (Figure C1 and C2). There is a clear N1-P2 complex at stimulus onset (Figure C1), whereas we see an additional divergence at the NP2 onset (Figure C2). It thus seems that the NP2 onset is processed differently than the stimulus onset, suggesting that the boundary was perceived as such.

# Appendix D: Exploratory analysis of latency differences

After visual inspection of the waveforms (Figure 3 in the main text), we realised that the waveforms were shifted in time due to the presence or absence of the final lengthening cue in *Lilli.* As a result, the boundary itself occurred in different moments in time across conditions. We decided to explore the effect of changing our time-locking point from the onset of the second syllable of *Lilli* to the offset of *Lilli*. This allowed us to align all boundaries at exactly the same position (Figure D1).

**Figure D1**

*ERP waveforms, time-locked to preboundary syllable offset*


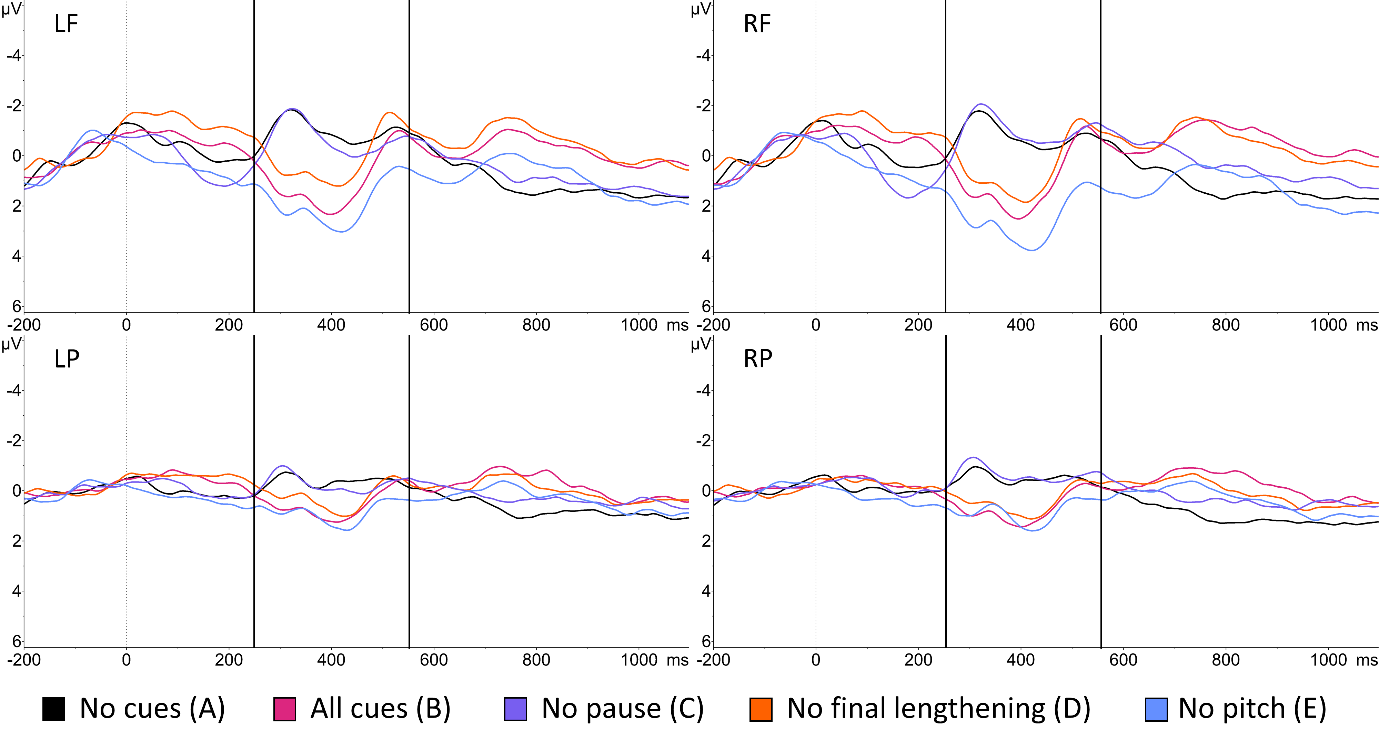


*Note.* ERP waveforms are low-pass filtered at 8Hz for illustration purposes. Upper left: left frontal; upper right: right frontal; lower left: left posterior; lower right: right posterior. Vertical dotted line: offset of the preboundary syllable. Vertical solid lines correspond to the analysed time-window.

As also found by Bögels et al (2010), by moving the time-locking point closer to the boundary, the CPS became more focal (Figure D1). Changing the timelock from preboundary syllable onset to preboundary syllable offset, the CPS should now occur in the time-window 250-550 ms after the offset of *Lilli.* Using the mean amplitude in this new time-window, we redid our main analysis with the same contrasts as reported in the main analysis. The results were as follows:

Boundary conditions compared to the no-boundary condition were more positive in all regions (LF: β = 1.60 µV, *SD* = 0.28, *t* = 5.649, *p* < 0.001; RF: β = 1.59 µV, *SD* = 0.28, *t* = 5.612, *p* < 0.001; LP: β = 0.73 µV, *SD* = 0.28*, t* = 2.583, *p* = 0.016; RP: β = 0.84 µV, *SD* = 0.28, *t* = 2.953, *p* = 0.006). This finding did not change from the results of our main analysis.

Compared to the all-cues condition, leaving out the pause cue resulted in a decrease positivity across all regions (LF: β = -1.78 µV, *SD* = 0.32, *t* = -5.540, *p* < 0.001; RF: β = -2.04 µV, *SD* = 0.32*, t* = -6.338, *p* < 0.001; LP: β = -0.88 µV, *SD* = 0.32, *t* = -2.730, *p* = 0.011; RP: β = -1.33 µV, *SD* = 0.32, *t* = -4.146, *p* < 0.001). This finding did not change from the results of our main analysis.

Compared to the all-cues condition, leaving out the final lengthening cue resulted in a significant decrease in amplitude in the LF region (β = -0.97 µV, *SD* = 0.35, *t* = -2.776, *p* = 0.009). There were no significant differences in any of the other regions (RF: *p* = 0.10; LP: *p* = 0.31; RP: *p* = 0.43). This finding is different from the results of our main analysis.

Compared to the all-cues condition, leaving out the pitch rise cue resulted in an increase in positivity in the frontal regions (LF: β = 0.82 µV, *SD* = 0.27*, t* = 3.045, *p* = 0.004 and RF: β = 1.47 µV, *SD* = 0.27, *t* = 5.468, *p* < 0.001). There were no significant differences in the posterior regions (*p* = 0.31 and 0.51 for left and right respectively). This finding is different from the results of our main analysis.

Looking at the waveforms in Figure D1, we found an explanation for the differences found for leaving out final lengthening and leaving out pitch. The no-pitch waveform and the no-lengthening waveforms look almost identical in morphology to the waveform of the all-cues condition. This suggest that at least the same cognitive processes are occurring in both conditions. However, by shifting the time-locking point, the remaining boundary cues now occur during the baseline. This contaminates the baseline, which is otherwise supposed to be identical across conditions. As a consequence, it is possible that the signal had different mean values across conditions, shown by the three waveforms already starting to diverge right after the baseline.

Instead of changing the time-lock point, we also attempted to do a more heuristic exploratory analysis on peak amplitude, which should allow us to compare amplitudes across conditions independent of its latency. Using BrainVision Analyzer, we determined global peaks in the time-window 500-800 ms per participant automatically. Because we saw in Figure 3 (in the main text) that the maximum peak for no-boundary and no-pause conditions were negative in polarity, global minima were determined whereas global maxima were determined for the other boundary conditions. With the same contrasts and model as in our main analysis, we found the following for peak amplitude:

Boundary conditions compared to the no-boundary condition were more positive in all regions (LF: β = 5.90 µV, *SD* = 0.29*, t* = 20.605, *p* < 0.001; RF: β = 5.79 µV, *SD* = 0.29, *t* = 20.239, *p* < 0.001; LP: β = 4.30 µV, *SD* = 0.29, *t* = 15.036, *p* < 0.001; RP: β = 4.25 µV, *SD* = 0.29, *t* = 14.865, *p* < 0.001). This finding was consistent with the results of our main analysis.

Compared to the all-cues condition, leaving out the pause cue resulted in a decrease in peak amplitude in all regions (LF: β = -8.11 µV, *SD* = 0.47*, t* = -17.227, *p* < 0.001; RF: β = -8.35 µV, *SD* = 0.47, *t* = -17.726, *p* < 0.001; LP: β = -5.97 µV, *SD* = 0.47, *t* = -12.672, *p* < 0.001; RP: β = -6.33 µV, *SD* = 0.47, *t* = -13.449, *p* < 0.001). This finding was consistent with the results of our main analysis.

Compared to the all-cues condition, leaving out the final lengthening cue did not result in significant changes in peak amplitude (*p* = 0.37, 0.61, 0.98, and 0.55 in LF, RF, LP, and RP respectively). This is also the case for leaving out the pitch cue compared to the all-cues condition (*p* = 0.98, 0.30, 0.52, and 0.19 in LF, RF, LP, and RP respectively. These findings were consistent with the results of our main analysis.

Together, these findings suggest that despite the lengthening differences in our stimuli and latency differences in the observed responses, our main analysis was sufficiently accurate in establishing the CPS in the boundary conditions.
